# MicroRNAs as non-invasive biomarkers of renal disease

**DOI:** 10.1093/ndt/gfz183

**Published:** 2019-09-20

**Authors:** Katie L Connor, Laura Denby

**Affiliations:** Centre for Cardiovascular Science, Queen’s Medical Research Institute, University of Edinburgh, Edinburgh, UK

With an estimated global prevalence of chronic kidney disease (CKD) of 11–13%, non-invasive biomarkers of renal pathology are desperately required to enhance early diagnosis, guide prognosis and monitor response to treatment [1]. Currently, the mainstay of renal functional monitoring remains measurement of blood urea, serum creatinine and urinalysis, with renal biopsies adopted when diagnostic clarity is required.

The ideal biomarker should be non-invasive, stable and sensitive, and should dynamically and specifically reflect disease pathology so as to guide diagnosis, treatment response and prognosis. Since their discovery in 1993, the small (20–22 nucleotides) non-coding RNAs microRNAs (miRNAs) have been shown to have many of these qualities. MiRNAs in multiple renal diseases have now been explored in renal tissue and biofluids including blood, urine (whole, cell free and urinary pellet), serum, plasma and perfusate from renal *ex vivo* perfusion systems. The stable detection of miRNAs in many of these samples is enhanced by their binding to proteins (e.g. Argonaute and lipoproteins) and/or their encapsulation in extracellular vesicles—both of which limit their degradation by RNases. In addition to their role as biomarkers, their ability to negatively regulate key networks of genes by post-transcriptional repression of mRNAs has resulted in critical mechanistic roles being identified in development, homeostasis and disease in multiple organs including the kidney [2]. Here, we discuss promising non-invasive miRNA biomarkers of renal disease in humans, highlighting the opportunities, challenges and approaches that may enhance clinical translation of miRNA biomarkers.

The current classical pipeline for miRNA biomarker development is firstly high-throughput measurement of miRNAs by small-RNA sequencing or microarray in a discovery cohort containing the disease and sample of interest, followed by validation of key miRNAs in large and ideally prospective cohort studies by quantitative real-time PCR (qRT-PCR). Although miRNAs can be readily detected from a limited RNA quantity by qRT-PCR, the output from high-throughput sequencing often requires a greater RNA quantity that can often only be reliably obtained from renal tissue. However, the expression of many miRNAs initially profiled in renal tissue is often not faithfully reflected within contemporaneous biofluids. A further technical challenge in biofluid miRNA quantification is the normalization of miRNA expression levels to an exogenous and/or endogenous control. This is particularly important when quantifying miRNAs in biofluids such as urine that may drastically vary in concentration. At present, there is little consensus in the field on the optimal normalization approach and this may contribute to incongruous results between studies [3].

With their ability to regulate and reflect disease-specific molecular pathways, a potential advantage of miRNAs is that they may non-invasively offer diagnostic discrimination over generic biomarkers of renal injury. For example, microRNA-148b (miR-148b) and miR-let-7b have been shown experimentally to regulate the O-glycosylation process of immunoglobulin A1 (IgA1) in the peripheral blood mononuclear cells of patients with IgA nephropathy [4]. Subsequently, Serino *et* *al.* conducted a large international study and demonstrated that serum miR-148b and miR-let-7b could discriminate patients with IgA nephropathy from both controls and patients with other forms of glomerulonephritis [5]. While a number of studies can clearly distinguish patients with established renal disease from healthy controls, it is the ability to both detect disease at the early stages and discriminate the disease of interest from other renal pathologies where most diagnostic miRNA signatures require further validation.

Another potential clinical utility of miRNAs is as predictive markers. Within acute kidney injury (AKI), a key unmet need is to develop a biomarker that predicts the risk of progression to severe AKI. MiR-21 remains one of the most extensively studied miRNAs, in the context of ischaemic AKI upregulation of urinary and to a lesser extent plasma miR-21 predicted AKI progression in patients undergoing cardiac surgery [6]. In transplantation, miR-21 was found to be upregulated in the first urine passed of patients with delayed graft function, with the accuracy of this biomarker performing best when measured as a panel with six other miRNAs [7]. Incorporation of these miRNA targets with clinical variables identified from logistic regression in the former study importantly yielded the best predictive model.

In the context of CKD, another large study examined miR-126 and miR-223 in the serum of 601 CKD patients (Stages 1–5) with follow-up for 6 years [8] and found that both miRNAs decreased as compared with healthy controls. Although both miRNAs tended to be lower with advancing stages of CKD, there was no association with initial miRNA expression, and after correction for estimated glomerular filtration rate there was no association with loss of renal function or mortality, cardiovascular disease and renal-related events. As the field of renal miRNA biomarkers develops and large well-conducted studies demonstrating non-overlapping results are reported, meta-analyses may be required. This was the approach utilized by Park *et* *al*., allowing them to conclude that miR-126 and the miR-770 family in urine and blood were the most promising markers to predict progression of diabetic nephropathy [9].

An attractive use of miRNAs would be as treatment modifiable biomarkers to assess response to treatment. In patients with hypertensive nephropathy (HN), miR-103a-3p was upregulated as compared with healthy controls in urine and serum. Mechanistically, the authors demonstrated that miR-103a-3p contributed to angiotensin II-induced HN via the SNRK/nuclear factor-κB/p65 regulatory axis [10]. Interestingly, patients treated with an angiotensin-converting enzyme inhibitor or β-blocker who responded positively with a reduction in their albumin–creatinine ratio demonstrated a fall in miR-103a-3p, suggesting that this miRNA may dynamically reflect disease pathology and treatment response.

A number of promising non-invasive miRNA biomarkers have now been identified in renal disease with the potential to augment diagnosis, guide prognosis and monitor response to treatment—just a few are highlighted here (Figure 1). Large prospective clinical studies with heterogenous case mix and long follow-up are now urgently needed to determine the accuracy and generalisability of these markers. It appears more likely that any clinical use will require a panel of miRNAs or miRNAs used in combination with other biomarkers and clinical scores to improve diagnostic utility. In addition, more meta-analyses are likely to be required due to multiple studies with non-overlapping results.

**FIGURE 1:**
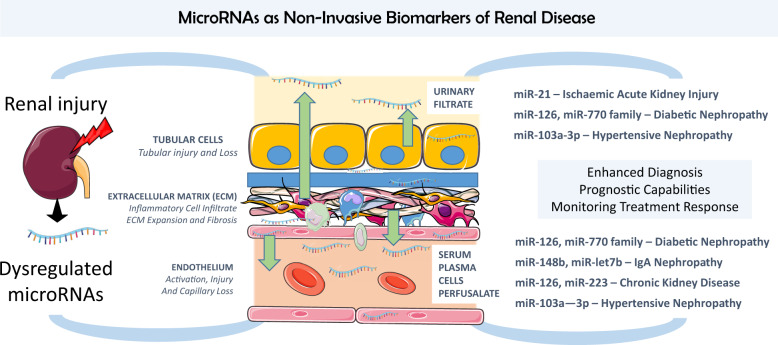
Opportunities and sources of non-invasive microRNA biomarkers in renal diseases. MicroRNAs highlighted in the article are schematically drawn here to depict source and biomarker potential. Made using Servier Medical Art.

## FUNDING

The authors are supported by funding from the Medical Research Council (MR/S001743/1) and Kidney Research UK (SF_001_20181122;RP_042_20160304).

## CONFLICT OF INTEREST STATEMENT

L.D. is an awardee of PhD studentships co-funded by Regulus Therapeutics and GSK.
